# Relationship between Lower Urinary Tract Symptoms and Treatment-Related Behavior in an Eastern European Country: Findings from the LUTS POLAND Study

**DOI:** 10.3390/ijerph18020785

**Published:** 2021-01-18

**Authors:** Mikolaj Przydacz, Przemyslaw Dudek, Tomasz Golabek, Piotr Chlosta

**Affiliations:** Department of Urology, Jagiellonian University Medical College, 30-688 Krakow, Poland; przemekdudek@op.pl (P.D.); elementare@op.pl (T.G.); piotr.chlosta@gmail.com (P.C.)

**Keywords:** Poland, LUTS, OAB, epidemiology, treatment seeking

## Abstract

**Background:** The aim of this study was to investigate the effect of lower urinary tract symptoms (LUTS) on behavior related to treatment of Polish adults aged ≥ 40 years. **Methods:** We conducted a computer-assisted telephone survey with a study sample stratified by age, sex, and place of residence (type, size, urban versus rural) reflecting the entire Polish population. Participants rated the frequency and symptom-specific bother of individual LUTS and their effects on seeking and receiving treatment, treatment satisfaction, and treatment continuation. We adjusted multiple logistic regression models to analyze the simultaneous effects of predictor variables on each dependent variable. **Results:** Overall, 6005 participants completed the interview. One third (29.6–33.5%) of participants with LUTS were seeking treatment, and 24.0–26.4% received treatment. There was no difference in treatment seeking and receiving between urban and rural areas. Whereas storage and voiding symptoms were significantly related to treatment seeking by both men and women, treatment receiving correlated only with voiding symptoms in men and only with storage symptoms in women. Most respondents who received treatment were satisfied; treatment dissatisfaction was related to the presence of storage symptoms in both men and women. Only 50% of all participants continued their treatment; discontinuation of treatment was statistically more prevalent for women than for men. **Conclusion:** This investigation, the first population-representative study performed in Eastern Europe, revealed a low frequency of seeking treatment for LUTS. In addition, symptoms that inclined participants to seek treatment might not have been adequately addressed by the treatment they received. We also found a relatively high rate of treatment discontinuation. Clearly, there is a need for both improved patient education about LUTS treatment and a need for increased clinician awareness of the coexistence of different symptoms in men and women plus proactive evaluation by physicians for all types of LUTS and associated bother.

## 1. Introduction

Lower urinary tract symptoms (LUTS) include storage, voiding, and post-micturition symptoms [1]. The prevalence of LUTS has been reported in some large-scale population-based analyses. LUTS were found to affect up to 74% of adults aged ≥ 40 years in Europe and North America [2], 75% in South America [3], and 61% in Asia [4]. In a recent population-representative epidemiological study of LUTS in Poland, the first reliable and nationwide epidemiological study of LUTS in an Eastern European country, we reported LUTS prevalence of 69.8% in adults aged ≥ 40 years, with more women affected than men (72.6% vs. 66.2%) [5].

Although not considered life-threatening, LUTS can be highly bothersome and can negatively affect social functioning, mental health, sleep, sexuality, productivity, and overall quality of life [6]. However, individuals have many concerns that prevent them from seeking help. Studies of barriers to seeking treatment have pointed to embarrassment, anxiety, social stigma, or acceptance of LUTS as an unavoidable aspect of aging [7]. Often, people are simply unaware that there may be treatment for their ailment [8]. It is important to know which LUTS and symptom-related bothers prompt men and women to seek treatment because these data would be valuable in adjusting treatment approaches. This knowledge may also support health improvement programs, educational campaigns, and resource allocation.

Whereas healthcare-related behaviors for LUTS have been investigated in Western Europe and some other regions of the world, there are no data for countries of Central and Eastern Europe [8]. Moreover, most studies that have examined the effects of symptoms and symptom-specific bother on healthcare-related behaviors have been conducted for patient-based samples; data for the general population are especially sparse, regardless of the region of the world. Because some local cultural norms such as lifestyle factors may inhibit individuals from admitting or discussing their health issues, the healthcare-related behaviors may also vary between countries and regions [2,9]. Indeed, Central-Eastern Europe is often considered a distinct cultural entity [10]. As Slavic people, Poles are culturally different from other European people, particularly Germanic and Romance people [11]. Additionally, with a relatively high number of people living in Polish rural regions, available data on behavior related to LUTS treatment may not be fully transferable to Poland because relationships between LUTS and treatment-related behavior have not been reported and compared between urban and rural areas. Therefore, the aim of this study was to analyze the effect of LUTS on treatment seeking, treatment receiving, treatment satisfaction, and treatment continuation, in a representative group of men and women aged ≥40 years in Poland, the largest country by land area in Central Europe and the third most populous country in Eastern Europe [12,13,14]. 

## 2. Materials and Methods

This was a population-based and cross-sectional study performed in Poland. The goal was to examine the prevalence and symptom-specific bother of LUTS and to evaluate the impact of these symptoms on behavior related to treatment. The study included representative pools of men and women aged ≥ 40 years living in all geographical regions of Poland (including urban and rural areas with appropriate proportions). We described the justification for this recruitment approach and the details of the survey and study design elsewhere [5]. The Ethics Committee of Jagiellonian University Medical College, Krakow, Poland (1072.6120.160.2019) approved the study. The study is registered with ClinicalTrials.gov (NCT04121936).

A computer-assisted telephone survey was conducted between 1 September and 30 December 2019. We used the most recent census and a sample-matching method to create a target sample [15]. Either before or after completion of the questionnaires, we stratified the survey sample by age, sex, and place of residence (for both geographical regions, i.e., 16 states = voivodships, and type/size of living places, including an adequate proportion of urban and rural areas) to reflect the entire Polish population. We excluded participants with current/past urinary tract infection (within one month), women pregnant at the time of the survey, and women who had given birth within the preceding six months.

All participants reported the demographics and presence of LUTS, as recommended by the International Continence Society (ICS), which encompassed frequency, urgency, urgency with fear of leaking, nocturia, urinary incontinence (urgency, stress, mixed, leak for no reason), intermittency, slow stream, splitting/spraying, hesitancy, terminal dribble, straining, incomplete emptying, and post-micturition dribble [1]. The International Prostate Symptom Score (IPSS) [16] and the Overactive Bladder-Validated 8-question Screener (OAB-V8) [17] were also used. All terms and instruments were adapted, validated, and presented in Polish. 

Participants graded the frequencies of experiencing individual LUTS during the prior month; ratings were based on a Likert-like scale: none (score 0), less than 1 in 5 times (score 1), less than half the time (score 2), about half the time (score 3), more than half the time (score 4), or almost always (score 5). For frequencies of at least “less than 1 in 5 times”, participants were also asked about the degree of associated bother by the particular LUTS and scored: not at all (score 0), a little bit (score 1), somewhat (score 2), quite a bit (score 3), a great deal (score 4), or a very great deal (score 5). Table 1 enumerates the telephone survey questions pertaining to treatment seeking, treatment receiving, satisfaction, and continuation.

To make our results comparable to other epidemiological analyses of LUTS, we used two definitions for LUTS prevalence: definition I, symptoms occurring less than half the time or more, and definition II, symptoms occurring half the time or more [2,3]. 

### Statistics

We used the Pearson chi-squared test to assess correlations of categorical variables. We adapted several logistic regression models to measure concurrent effects of predictor variables on dichotomous dependent variables (treatment seeking, treatment receiving, satisfaction, and continuation). The predictor variables we considered were age, sex, education, employment, marital status, urban or rural residence, frequency, and bother of each LUTS. For regression analysis, occurrence of a particular LUTS was considered established when the LUTS was experienced less than half the time or more (i.e., IPSS grade ≥ 2); bother was considered to exist when it was rated somewhat or greater (equal to OAB-V8 grade ≥ 2).

The group of variables was large; thus, we chose predictor variables for each model when the association of the predictor variables with the dependent variable reached 20% significance in univariate analysis. Initially, we included all selected variables; then, we sequentially eliminated variables with < 5% significance (except for age, the control variable) in the order of their significance (backward method). We separately adjusted models for men and women. All the data were analyzed with SPSS Statistics software (IBM Corporation, Armonk, NY, USA, version 24.0).

## 3. Results

### 3.1. Treatment Seeking and Treatment Receiving

Among a group of respondents with symptoms that occurred less than half the time or more (definition I), 29.6% (*n* = 1239) were seeking treatment and 24% (*n* = 1004) received treatment (Table 2). 

For respondents with symptoms that occurred half the time or more (definition II), 33.5% (*n* = 1013) were seeking treatment, and, again, many of these persons received treatment (26.4%; *n* = 800). On the basis of definition II, we found that statistically more men than women sought and received treatment (Table 2). This trend was also hinted at with definition I; however, it was not statistically significant. We did not find differences in looking for treatment and treatment received between individuals who resided in urban and rural areas. 

Men with bother associated with urgency, frequency, slow stream, hesitancy, and incomplete emptying were more likely to seek treatment compared with men without these symptoms (Table 3). 

Women bothered by urgency, frequency, urgency urinary incontinence, intermittency, and straining were more likely to seek treatment than women without these symptoms. We investigated an increase in the likelihood of treatment seeking for LUTS with each additional year of age for both sexes; for men, an increase of 7% in seeking treatment with each successive year and, for women, an increase of 3% per year (*p* < 0.01). For men, the chance of seeking treatment for LUTS was higher for persons married or living with a partner. 

The most frequent treatment was prescription drugs (definition I: 70%; definition II: 69.4%) followed by over-the-counter drugs (26.1%; 23%), physiotherapy (19.6%; 19.9%), surgery (17%; 18.3%), and lifestyle changes (14.7%; 15.3%). One-third of respondents (definition I: 32.4%; definition II: 32.1%) received combined treatment, i.e., at least two of the aforesaid treatment methods. 

Men bothered by intermittency, straining, and incomplete emptying were more likely to have been receiving treatment compared with men who lacked the symptoms (Table 3). Women with urgency with fear of leaking and bothersome frequency were more likely to receive treatment relative to women without those symptoms. Both men and women experienced yearly increases in the likelihood of receiving treatment for LUTS (an increase of 10% per year for men and 6% for women; *p* < 0.01).

### 3.2. Treatment Satisfaction

Significantly more men than women were satisfied with the treatment they received (definition I: 85.7% vs. 78.9%; definition II: 79.9% vs. 72.1%; Table 2). Consequently, more women than men were dissatisfied with the treatment (definition I: 21.1% vs. 14.3%; definition II: 27.9% vs. 20.1%). There was no influence of urban versus rural status on treatment satisfaction. 

Men bothered by urgency incontinence, urgency with fear of leaking, and frequency and women bothered by urgency incontinence, urgency with fear of leaking, frequency, and incomplete emptying had a higher risk of treatment dissatisfaction compared with individuals lacking these symptoms (Table 3). For women, there was a 5% yearly reduction in risk of treatment dissatisfaction (*p* < 0.01).

### 3.3. Treatment Continuation

More men than women reported continuation of treatment (definition I: 75.3% vs. 35%; definition II: 76.1% vs. 38.6%; Table 2); thus, women were more likely to discontinue treatment (definition I: 65.0% vs. 24.7%; definition II: 61.4% vs. 23.9%). The decision to continue or discontinue treatment was not affected by urban versus rural status.

Men with slow stream and bothersome post-micturition dribble had a lower risk of treatment discontinuation compared with men lacking those symptoms (Table 3). Conversely, men with incomplete emptying had a higher risk of stopping treatment. Women bothered by leak for no reason were at greater risk of treatment cessation compared with women without this symptom. 

### 3.4. Combination Relationships

A combination of treatment satisfaction and treatment continuation was statistically more prevalent in men than women, regardless of the LUTS prevalence definition (Table 2). Conversely, women were more likely to discontinue their satisfied treatment (definition I: 51.5%) compared with men (definition I: 23.4%). Dissatisfied women were also more likely to stop their treatment (definition I: 13.5%) compared with men (definition I: 1.4%).

## 4. Discussion

This investigation is the first large population-based study performed in Eastern Europe that analyzed the scope of patient preferences for treatment for LUTS. The study included all geographical regions of Poland, urban and rural, and provides reliable, valid, and consistent information of LUTS prevalence and behavior related to treatment. Although LUTS were highly prevalent in individuals aged ≥ 40 in Poland, less than one-third of people affected by LUTS pursued treatment.

The low percentage of people with LUTS who sought treatment has been reported elsewhere. The Epidemiology of Lower Urinary Tract Symptoms (EpiLUTS) study, an Internet-based population inquiry in Sweden, the USA, and the UK determined that only 29% of men and 28% of women affected by LUTS sought treatment for bladder problems [8]. In Asia, an Internet survey with participants from South Korea, Taiwan, and China showed that 26% of respondents with any LUTS reported visiting healthcare professionals because of their LUTS [4]. The Brazil LUTS, a telephone interview conducted in five major cities of Brazil, documented that up to 30.6% of respondents with LUTS sought treatment [18]. Our observation of treatment seeking by 29.6% of adults in Poland who reported LUTS is broadly comparable with the foregoing population-based studies. Therefore, the poor attention to health by individuals with LUTS is a significant, global concern.

We need to reflect on several shared pathways that restrain people from seeking help. First, LUTS can cause embarrassment, thereby inhibiting the search for medical help. Qualitative study findings suggest that these issues are worsened by apprehension even to seek reassuring environments to discuss delicate matters with healthcare professionals [19]. Second, there is still considerable belief that LUTS are a “natural” part of aging. People with LUTS may be perceived by themselves and by their partners and families as being frail and aged [7]. Third, the public is mostly unaware that LUTS can be treated with consequent improvement in the quality of life [20]. Some people consider LUTS to be incurable or untreatable, whereas others have concerns about the financial costs or the adverse effects of treatment (e.g., side effects of medication) [21]. Without adequate information about treatment, individuals cannot take optimal action for treatment seeking. Clearly, education is a crucial factor for treatment seeking. An individual’s understanding of LUTS can affect motivation and adherence, which can influence the treatment outcome [22]. Importantly, education and counseling of LUTS can be provided by a variety of clinicians, such as physicians (including multiple professions, e.g., urologists, gynecologists, general practitioners, geriatrists), nurses, and, in some cases, physiotherapists specialized in pelvic floor physiotherapy.

In our study, bother related to symptoms in distinct categories, i.e., storage (for men: frequency, urgency; for women: frequency, urgency, urgency urinary incontinence), voiding (for men: slow stream, hesitancy; for women: intermittency, straining), and post-micturition (for men: incomplete emptying), was correlated with a higher likelihood of seeking treatment. These data support the concept that voiding symptoms are not the only LUTS of men and that storage symptoms are not the only LUTS of women because men and women looked for treatment regardless of the LUTS subgroup. Therefore, the norm in routine clinical practice should be a broad and symptom-driven approach wherein LUTS are not disease- or condition-specific. In addition, despite being commonly related to bladder outlet obstruction, LUTS may be indicative of bladder dysfunction and other structural and/or functional abnormalities of the urinary tract; therefore, LUTS may herald many non-urological conditions [23]. Thus, for effective and individualized treatment, patients with symptoms from multiple categories need extensive and thorough diagnostic evaluation with a holistic approach for their LUTS. We found that more men than women sought treatment for LUTS, a phenomenon that coincided with other international studies that reported men were more likely to initiate conversations with clinicians [24,25]. Nevertheless, clinicians still need a proactive attitude toward both men and women. 

Most participants seeking treatment received it. For men, only intermittency, straining, and incomplete emptying were correlated with relatively greater reception of treatment. However, these symptoms are only voiding and post-micturition that physicians commonly associate with benign prostatic hyperplasia. As both frequency and urgency were related to treatment seeking by men, and these symptoms did not correlate with receiving treatment, storage symptoms may represent an unmet need. Conversely, in women, only those with urgency with fear of leaking and frequency were more likely to receive treatment, even though intermittency and straining were related to treatment seeking. Therefore, it appears that physicians considered only storage symptoms in their treatment plans for women. Thus, we hypothesize that not only patients but also clinicians, non-urologists in particular, should be adequately educated about LUTS diversity and coexistence, their comprehensive evaluation, and various treatment methods. 

These unmet needs have additional implications. We found that men with bother from frequency, urgency with fear of leaking, and urgency incontinence were more likely to be dissatisfied with their treatment. Because storage symptoms in men correlated with treatment seeking but were not associated with receiving treatment, persisting with ineffective/inadequate overall management in men may have a profound negative impact on treatment satisfaction. Control of storage symptoms is often unsatisfactory in men with treatment only for voiding symptoms, i.e., with alpha-blockers or 5-alpha-reductase inhibitors. Anticholinergics or agonists of the beta-3 receptor are recommended as a first-line therapy for storage symptoms associated with overactive bladder. However, clinicians may refrain from prescribing antimuscarinics because of unfounded notions that they promote urinary retention [26]. The European Association of Urology recommends a combination of an alpha-blocker with an antimuscarinic for men with moderate or severe LUTS, provided that risk factors for progression of benign prostatic hyperplasia are absent and that alpha-blocker monotherapy is insufficient to relieve storage symptoms [23].

For women, treatment dissatisfaction was statistically higher for urgency with fear of leaking and urgency urinary incontinence, although storage symptoms correlated with both treatment seeking and treatment receiving. This difference may result from storage symptoms being poorly controlled. In instances of ineffective treatment, the European Association of Urology recommends considering dose escalation or offering an alternative antimuscarinic formulation, or mirabegron, or a combination [27]. Notably, in recent international studies, mirabegron was associated with a significantly longer time to discontinuation, greater persistence, and better adherence than achieved with antimuscarinics [28]. Treatment dissatisfaction of women also correlated with incomplete emptying, which may be one of the symptoms of pelvic organ prolapse. This finding may further highlight that LUTS are not related to only dysfunctions of the bladder–sphincter complex. Unsatisfied treatment for LUTS requires thorough investigation of LUTS origin and sometimes close cooperation between urologists and gynecologists. 

Patients discontinue LUTS treatment at high rates [28]. In our study, statistically more women than men discontinued their treatments. This observation may be explained in two different ways. Whereas treatment for LUTS in men typically includes alpha-blockers or 5-alpha-reductase inhibitors with mild side effect profiles, treatment of LUTS in women often includes anticholinergics, with significant dropouts because of adverse events [29]. Alternatively, until now, public urological campaigns in Poland have been focused mainly on men, and women might be inadequately targeted. Thus, we speculate that men in Poland may be better educated about LUTS. Regardless, in Poland, future health-improvement programs in functional urology must reach men and women. 

Increasing age had effects on treatment seeking and treatment receiving by men and women, but this correlation was more evident for men (7–10% increase with each additional year of age) than women (3–6% yearly increase). We also found a 5% reduction in treatment dissatisfaction by women for each additional year of age, but no such trend was evident for men. Corresponding findings have been reported in some previous studies [8,24]. Some experts account for this difference by noting that women usually start earlier than men with a routine of frequent healthcare visits [18].

We did not find differences between urban and rural status in treatment-related behaviors. Before conducting the study, we hypothesized that people from urban areas would be more active in treatment seeking compared with people from rural regions. This hypothesis emerged from a report that people in rural areas in Poland were more hesitant to admit or discuss their health issues [30]. However, the data for that conclusion are decades old. Since 2004, Poland has been a member of the European Union, the organization that initiated and funded several large health improvement programs in Polish rural areas [31]. Even without longitudinal analyses of this correlation, with our results, we hypothesize that the health differences between rural and urban areas in Poland are beginning to blur. Further, the growing population density in Poland is leading to the merging of urban and rural areas [31].

This study was limited by the fact that persons self-reported LUTS and treatment. Cold-calling particularly limits assessment of treatment continuation and discontinuation. This concern is important with respect to patients who received surgical treatment. Although surgical treatment for LUTS (e.g., implantation of midurethral sling in women or transurethral resection of prostate in men) is successful in most cases, some patients may not benefit from surgery, and surgical treatment for LUTS may not be definitive. Further, some treatment options may be adjusted after surgery (e.g., reprogramming of pulse generator after electrode implantation in sacral neuromodulation) or treatments may require repetition (e.g., injections of onabotulinumtoxinA or bulking agents). We must also remember that some patients treated with surgery may also receive other treatments and they might continue or discontinue the nonsurgical interventions. With a cross-sectional design, at the time of interview, some respondents might have been awaiting scheduled surgery. Therefore, we could not rigidly qualify patients who received surgery as either continuing or discontinuing treatment; instead, we asked the respondents an open question regarding their overall treatment status (i.e., continuation or discontinuation). Contrary to clinic-based studies, estimation of treatment continuation or discontinuation for LUTS at the population level is more difficult, and there is no perfect standard measure for this parameter in studies such as ours. Nonetheless, the approach we adopted has been used in other large-scale population-based studies that analyzed LUTS and treatment behaviors related to LUTS [3,4,8,32]. We designed our methodology to enable comparisons of our results with other populations. We needed to provide results for the entire population in order to make available estimates that attract interdisciplinary frameworks for national health improvement programs with appropriate allocation of resources by governments and healthcare systems. However, the need for population estimates required some generalizations (e.g., merging conservative, pharmacological, and surgical treatments). In addition, we did not collect data concerning barriers to healthcare-seeking and drug-related adverse effects. 

## 5. Conclusions

Although LUTS were highly prevalent in Poland, the degree of treatment seeking was low. The occurrence of symptoms from all three ICS symptom groups (storage, voiding, post-micturition) and the related bother were correlated with treatment-related outcomes, including treatment seeking, treatment receiving, treatment satisfaction, and/or treatment continuation. We observed these relationships with men and women. Clearly, there is a need to educate the general Polish population about potential benefits of LUTS treatment. In addition, it is incumbent upon physicians to proactively inquire with patients about LUTS and comprehensively assess multiple/various symptoms and, especially, associated bother.

## Figures and Tables

**Table 1:** Questions regarding treatment seeking, treatment receiving, treatment methods used, treatment satisfaction, and treatment continuation.

Symptoms: frequency, urgency, urgency with fear of leaking, nocturia, urinary incontinence (urgency, stress, mixed, leak for no reason), intermittency, slow stream, hesitancy, straining, splitting/spraying, terminal dribble, incomplete emptying, post-micturition dribble
1. Have you sought medical attention for your urinary or bladder problems?
Yes/No
2. Have you received any treatment for your urinary or bladder problems?
Yes/No
3. Which of the following methods of treatment did you use?
Lifestyle changes/Exercise and physiotherapy/Non-prescription drugs/Prescription drugs/Surgical treatment
4. Do you continue the treatment?
Yes/No
5. Are/Were you satisfied with the treatment?
Yes/No

**Table 2:** Treatment-related behaviors.

	Sex				Place of Living					
	Men		Women		Urban		Rural		Total	
	*n*	%	*n*	%	*n*	%	*n*	%	*n*	%
Men and women with symptoms occurring less than half the time or more (definition I) ^										
Treatment seeking	541	31.3	698	28.4	776	30.1	463	28.8	1239	29.6
Treatment receiving	441	25.5	563	22.9	634	24.6	370	23.0	1004	24.0
Treatment satisfaction	378 *	85.7	444	78.9	511	80.6	311	84.1	822	81.9
Treatment dissatisfaction	63	14.3	119	21.1	123	19.4	59	15.9	182	18.1
Treatment continuation	332 **	75.3	197	35.0	340	53.6	189	51.1	529	52.7
Treatment discontinuation	109 **	24.7	366	65.0	294	46.4	181	48.9	475	47.3
Treatment satisfaction with continuation	275 **	62.4	154	27.4	272	42.9	157	42.4	429	42.7
Treatment satisfaction with discontinuation	103 **	23.4	290	51.5	239	37.7	154	41.6	393	39.1
Treatment dissatisfaction with continuation	57	12.9	43	7.6	68	10.7	32	8.6	100	10.0
Treatment dissatisfaction with discontinuation	6 *	1.4	76	13.5	55	8.7	27	7.3	82	8.2
Men and women with symptoms occurring at least half the time (definition II) ^										
Treatment seeking	457 *	37.9	556	30.6	631	33.7	382	33.2	1013	33.5
Treatment receiving	373 *	30.9	427	23.5	497	26.5	303	26.3	800	26.4
Treatment satisfaction	298 *	79.9	308	72.1	367	73.8	239	78.9	606	75.8
Treatment dissatisfaction	75	20.1	119	27.9	130	26.2	64	21.1	194	24.2
Treatment continuation	284 **	76.1	165	38.6	282	56.7	167	55.1	449	56.1
Treatment discontinuation	89 **	23.9	262	61.4	215	43.3	136	44.9	351	43.9
Treatment satisfaction with continuation	230 **	61.7	124	29.0	217	43.7	137	45.2	354	44.3
Treatment satisfaction with discontinuation	68 *	18.2	184	43.1	150	30.2	102	33.7	252	31.5
Treatment dissatisfaction with continuation	54	14.5	41	9.6	65	13.1	30	9.9	95	11.9
Treatment dissatisfaction with discontinuation	21	5.6	78	18.3	65	13.1	34	11.2	111	13.9

^ Based on definition I, prevalence of LUTS was 69.8% (men: 66.2%; women 72.6%). Based on definition II, prevalence of LUTS was 50.4% (men: 46.2%; women: 53.5%). * *p* < 0.05 between men and women. ** *p* < 0.01 between men and women.

**Table 3:** Heat map of logistic and ordered multiple regressions for treatment seeking, treatment receiving, dissatisfaction, and discontinuation by men and women. Numbers show adjusted odd ratios (95% confidence interval). Red cells indicate an increase and blue cells indicate a decrease in the likelihood of treatment seeking, treatment receiving, dissatisfaction, or discontinuation. Blank cells indicate that there was not a statistically significant relationship.

Covariates	Treatment Seeking	Treatment Receiving	Treatment Dissatisfaction	Treatment Discontinuation
Men				
Storage symptoms				
Nocturia ^b^				
Nocturia ^b^—bother				
Frequency				
Frequency—bother	1.98 (1.04–3.61)		4.79 (1.32–10.24)	
Urgency				
Urgency—bother	2.11 (1.15–3.99)			
Urgency with fear of leaking				
Urgency with fear of leaking—bother			5.11 (1.76–11.23)	
Urgency urinary incontinence				
Urgency urinary incontinence—bother			5.99 (1.88–14.43)	
Stress urinary incontinence				
Stress urinary incontinence—bother				
Mixed urinary incontinence ^c^				
Mixed urinary incontinence ^c^—bother				
Leak for no reason				
Leak for no reason—bother				
Voiding symptoms				
Intermittency				
Intermittency—bother		2.01 (1.33–2.86)		
Slow stream				0.41 (0.21–0.97)
Slow stream—bother	2.29 (1.24–3.89)			
Hesitancy				
Hesitancy—bother	1.71 (1.12–2.65)			
Straining				
Straining—bother		2.42 (1.21–4.35)		
Splitting/spraying				
Splitting/spraying—bother				
Terminal dribble				
Terminal dribble—bother				
Post-micturition symptoms				
Incomplete emptying				6.11 (2.42–18.67)
Incomplete emptying—bother	2.93 (1.54–5.54)	3.45 (1.91–6.37)		
Post-micturition dribble				
Post-micturition dribble—bother				0.33 (0.18–0.91)
Demographics				
Age	1.07 (1.06–1.09)	1.1 (1.09–1.13)		
Educational status				
Work situation—unemployed				
Marital status	1.39 (1.01–1.89)			
Place of living (urban vs. rural)				
Women				
Storage symptoms				
Nocturia ^b^				
Nocturia ^b^—bother				
Frequency				
Frequency—bother	1.90 (1.19–2.72)	1.88 (1.27–2.91)	2.33 (0.99–4.98)	
Urgency				
Urgency—bother	2.24 (1.34–3.12)			
Urgency with fear of leaking		2.01 (1.41–3.11)		
Urgency with fear of leaking—bother		2.52 (1.74–3.81)	2.56 (1.04–5.32)	
Urgency urinary incontinence				
Urgency urinary incontinence—bother	2.31 (1.38–3.19)		2.78 (1.35–6.09)	
Stress urinary incontinence				
Stress urinary incontinence—bother				
Mixed urinary incontinence ^c^				
Mixed urinary incontinence ^c^—bother				
Leak for no reason				
Leak for no reason—bother				4.05 (2.03–10.11)
Voiding symptoms				
Intermittency				
Intermittency—bother	1.98 (1.14–3.58)			
Slow stream				
Slow stream—bother				
Hesitancy				
Hesitancy—bother				
Straining				
Straining—bother	1.85 (1.05–2.99)			
Splitting/spraying				
Splitting/spraying—bother				
Terminal dribble				
Terminal dribble—bother				
Post-micturition symptoms				
Incomplete emptying				
Incomplete emptying—bother			2.98 (1.44–6.99)	
Post-micturition dribble				
Post-micturition dribble—bother				
Demographics				
Age	1.03 (1.02–1.05)	1.06 (1.04–1.08)	0.95 (0.92–0.98)	
Educational status				
Work situation—unemployed				
Marital status				
Place of living (urban vs. rural)				

^b^ Nocturia was defined as two or more voids per night. ^c^ Participants who reported both urge and stress urinary incontinence symptoms were classified as having mixed urinary incontinence.
